# PGD2/PTGDR2 signaling pathway affects the self-renewal capacity of gastric cancer stem cells by regulating ATG4B ubiquitination

**DOI:** 10.3389/fonc.2024.1496050

**Published:** 2024-12-24

**Authors:** Qiang Zhang, HengJin Tian, Kunpeng Ge, FeiFan Wang, PeiYao Gao, AMin Chen, Lulu Wang, YanMing Zhao, Chaoqun Lian, FengChao Wang

**Affiliations:** ^1^ Department of Clinical Laboratory, The First Affiliated Hospital of Bengbu Medical University, Bengbu, China; ^2^ Key Laboratory of Cancer Research and Clinical Laboratory Diagnosis, Bengbu Medical University, Bengbu, China; ^3^ Department of Blood Transfusion, The First Affiliated Hospital of Naval Medical University, Shanghai, China; ^4^ Department of Clinical Laboratory, The Second People’s Hospital of Bengbu, Bengbu, China

**Keywords:** gastric cancer, cancer stem cells, PTGDR2, autophagy, ubiquitination

## Abstract

**Background:**

Prostaglandin D2 (PGD2) inhibits the development of different malignant tumors; however, the underlying mechanism of inhibiting tumor development is not yet clear. This study aimed to elucidate how PGD2 inhibits the stemness of gastric cancer stem cells (GCSCs) *via* autophagy and its underlying molecular mechanism to provide a theoretical basis for the treatment of gastric cancer.

**Methods:**

In this study, GCSCs were enriched *in vitro* by serum-free incubation. Furthermore, the effects of PGD2 and PGD2 receptor (PTGDR2) on autophagy were detected by Western blotting, immunofluorescence analysis, and transmission electron microscopy. Moreover, the ATG4B ubiquitination levels were assessed *via* immunoprecipitation and other methods.

**Results:**

The results indicated that PGD2 induced LC3I/LC3II conversion in GCSCs to activate autophagy, while PGD2 promoted the expression of PTGDR2, thereby further activating autophagy. Furthermore, PTGDR2 competes with ATG4B for binding with E3 ligase RNF5 (also known as RMA1) to promote autophagy protein ATG4B expression. Moreover, PTGDR2 knockdown blocked the activation of autophagy by PGD2 and the level of ATG4B ubiquitination in GCSCs.

**Conclusions:**

In summary, it was elucidated that the PGD2/PTGDR2 signaling cascade affects GCSCs stemness by regulating autophagy, suggesting that the PGD2/PTGDR2 signaling pathway could serve as a novel target for cancer therapy.

## Graphical Abstract

Schematic diagram of the study.

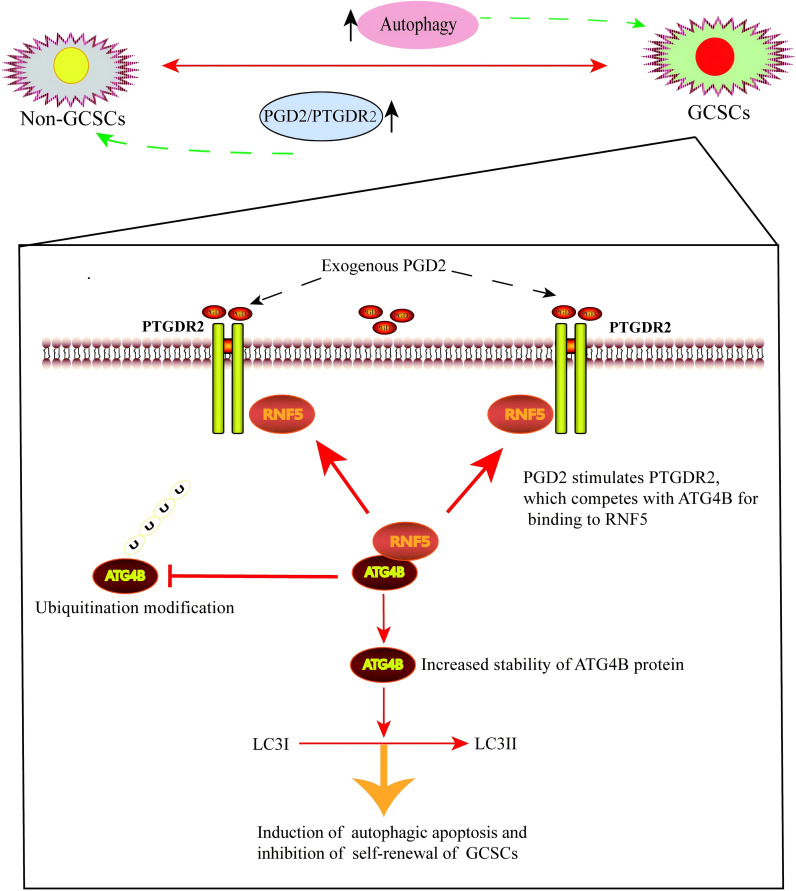

## 1 Introduction

Gastric cancer (GC) is a global health concern and one of the most malignant tumors with high morbidity and mortality. Since GC cells can metastasize, recur, and become resistant to radiotherapy and chemotherapy, therefore, the survival rate of advanced GC patients is very low, the mortality rate is high, and it is difficult to treat (1). Therefore, new therapeutic approaches to improve GC patient’s survival rate and quality of life are urgently required. Some researchers have indicated that cancer stem cells (CSCs) are a key factor responsible for the complex treatment of tumors. Furthermore, like normal stem cells, tumor stem cells have a strong self-renewal ability and can differentiate into heterogeneous tumor cells (2, 3). CSCs can xenograft tumors, and a small number of CSCs can induce tumorigenesis and are significantly associated with tumorigenesis and development (4). In addition, CSCs are highly plastic tumor cells, also known as lead cells, that cause chemotherapy/radiotherapy failure. Given the critical role of CSCs, new therapeutic approaches should be urgently explored for eradicating GC.

Some researchers have proposed that GCSCs originate from gastric tissues or bone marrow mesenchymal stem cells, and are responsible for the development of GC (5, 6). GCSCs, like CSCs, have highly expressed drug-resistance genes, which make them resistant to treatment and form the basis of drug resistance. Furthermore, transmembrane glycoprotein CD44 and octamer-binding transcription factor 4 (OCT4) are considered CSCs markers and can be used to isolate and characterize GCSCs as well as for targeted therapeutic studies for GC (7, 8).

PGD2 is a lipohormone-like signaling ligand for the enzymatic reaction of arachidonic acid (AA), a physiologically active lipid compound (9). The AA is broken down by cyclooxygenase into the intermediate product prostaglandin H2 (PGH2), which is finally converted to PGD2 catalyzed by L-PTGDS (10), the rate-limiting enzyme for PGD2 synthesis. Furthermore, PGD2 receptor 2 (PTGDR2) is one of the receptors for PGD2 (3). Moreover, the literature has indicated that L-PTGDS and PTGDR2 are expressed in GC tissues at reduced levels and are strongly associated with the prognosis of GC patients (3, 11). This study further demonstrated the critical role of the PGD2/PTGDR2 signaling cascade response in tumor biology. However, the molecular mechanisms of how the PGD2/PTGDR2 signaling pathway regulates the stemness of GCSCs need further elucidation.

Autophagy is a mechanism of intracellular homeostasis that promotes the degradation of unwanted components such as damaged proteins and organelles (12). Research studies have revealed that autophagy acts as a tumor suppressor by inhibiting the aggregation of damaged proteins or organelles (13). Furthermore, autophagy is also associated with the regulation of CSCs. Several studies have indicated that the activation of autophagy can decrease CSCs stemness. For example, Che et al. showed that lupeol activates autophagy, which reduces the self-renewal ability of the WERI-Rb-1 cell line (retinoblastoma) (14). Autophagy-associated 4B cysteine peptidase (ATG4B) is an autophagy regulatory molecule. It cleaves the LC3 protein to convert it to the cytoplasmic-associated form (LC3I), which is then activated by ATG7 to the autophagosome-associated form (LC3II), which is essential for autophagy activation (15). RNF5 (ring finger protein 5) is an E3 ubiquitin ligase that causes degradation of other proteins after ubiquitination. Furthermore, it promotes the ubiquitination of ATG4B, thereby degrading it *via* the proteasomal pathway and inhibiting the activation of autophagy. It has been demonstrated that PTGDR2 can bind RNF5 (16, 17). ATG4B is a potential biomarker of therapeutic response in CML stem/progenitor cells. Moreover, ATG4B can serve as a drug target in these stem cells (18). These studies suggest that the activation of autophagy plays an important role in the malignant biological functions of CSCs; however, the molecular mechanisms of autophagy activation remain elusive. This study assessed whether PGD2/PTGDR2 signaling regulates the self-renewal ability of GCSCs by activating autophagy, and also evaluated the molecular mechanisms underlying the activation of autophagy, to indicate if this signaling cascade can be used as a target for the treatment of cancer.

## 2 Materials and methods

### 2.1 Cell lines and cell culture

Human GC SGC-7901 and HEK-293T cell lines were purchased from Shanghai Cell Bank, Chinese Academy of Sciences, and cultured (RPMI)-1640/(DMEM) medium (Gibco, USA) augmented with 10% fetal bovine serum at 37°C and 5% CO_2_. Then the GCSCs (1×10^3^/well) were cultured in ultra-low adsorption 6-well plates in serum-free DMEM/F12 basal medium supplemented with B27 (10 μL/mL), bFGF (10 ng/mL), EGF (20 ng/mL). In each well 2 mL culture media was added and the cells were cultured for one week for further experiments.

### 2.2 Cell spheroid formation assay

Cells in the PBS and PGD2 (2.5, 5, and 10 μg/mL) groups were trypsinized, counted, and cultured at a density of 200 cells/well in ultra-low adsorption 6-well plates with 2 mL of serum-free suspension medium per well. After 7 days, the size and number of GCSCs in each group were assessed.

### 2.3 RNA extraction and RT-qPCR

Total RNA was extracted from GC cells using a total RNA isolation kit (Vazyme, Nanjing, China) and then subjected to reverse transcription for preparing complementary DNA. RT-qPCR was performed using 1 µg of total RNA and the ChamQ Universal SYBR qPCR Master MixKit (3). Primer sequences utilized in this study are listed in **Table 1**.

**Table 1:** Primer sequences.

Gene Symbol	Forward Primer 5’-3’	Reverse Primer 5’-3’
PTGDR2	TGCCTCTTGTCTAGCTGCTG	GACATCGTGGGGCTCTGG
CD44	CCTCTTGGCCTTGGCTTTG	CTCCATTGCCACTGTTGATCAC
OCT4	GGACCCAGGGAGAGACGTAA	CAUGGAUUUUUUGGAGCAGG
β-Actin	GACCTGTACGCCAACACAGT	CTCAGGAGGAGCAATGATCT

### 2.4 Western blot analysis

Total proteins were extracted from each group’s GC cells, lysed on ice, quantified, separated using SDS-PAGE, and then transferred to PVDF membranes. The membranes were then blocked for 2 h using milk, washed, and then treated with primary antibodies against OCT4, CD 44, ATG4B, RNF5, GAPDH and Ubiquitin (murine monoclonal). The membrane was then incubated with goat anti-rabbit IgG and goat anti-mouse IgG horseradish peroxidase-coupled secondary antibodies (Lianke Bio, China) for 2 hrs. The signals were detected by the Chemicalucent ECL detection system.

### 2.5 Immunofluorescence analysis

The cells were trypsinized and then centrifuged to acquire a single-cell suspension for culturing on confocal Petri dishes. After the cell’s attachment, they were fixed with pre-cooled methanol for 20 min, permeabilized with 0.3% Triton X-100 for 20 min, and then blocked using 1% BSA for 1 h. Then the cells were incubated with primary antibodies at 4°C overnight, washed three times with PBS, and then tagged with the secondary antibody at room temperature for 2 h. The nuclei were stained with DAPI for 2 min before observation using a microscope.

### 2.6 Cell transfection assay

Cell transfection with small interfering RNA (siRNA) was performed using lipofectamine 3000 (Invitrogen, USA). Control sequences and PTGDR2, RNF5 and si-ATG4B specific siRNA sequences are listed in **Table 2**.

**Table 2:** siRNA oligonucleotides.

Target Gene	Forward 5’-3’	Reverse 5’-3’
Si-NC	UUCUCCGAACGUGUCACGUTT	ACGUGACACGUUCGGAGAATT
Si-PTGDR2	UCAACACGGUGCCCUAUUUTT	AAAUAGGGCACCGUGUUGAGC
Si-RNF5	GGGGCCCCGAAGGGCCAAATT	UUUGGCCCUUCGGGGCCCCTT
Si-ATG4B	GAAGCUUGCUGUCUUCGAUTT	AUCGAAGACAGCAAGCUUCTT

### 2.7 Statistical analysis

The intergroup statistical differences were assessed by ANOVA and Student’s t-test using GraphPad Prism 8.0 statistical analysis software. All the data are expressed as mean ± standard deviation, with p<0.05 indicating statistically significant differences, and at least three trials were performed for each experiment.

## 3 Results

### 3.1 Culture and identification of GCSCs

**Figure 1:**
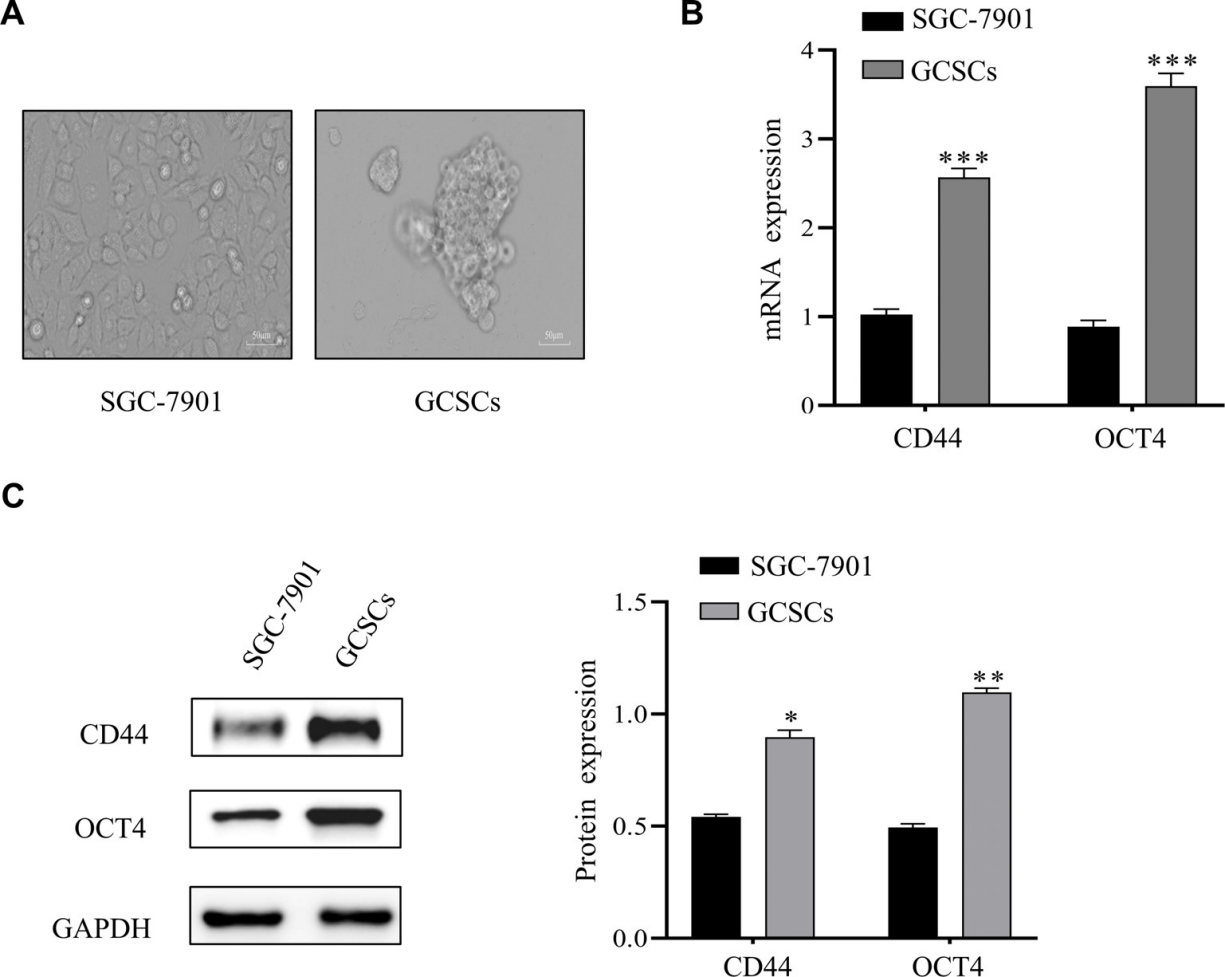
Culture and identification of GCSCs. **(A)** Images of serum-free cultured SGC-7901. **(B)** mRNA expression of CD44 and OCT4 in SGC-7901 cells and enriched GCSCs were detected by qRT-PCR. **(C)** Western blot analysis of stemness-associated protein levels in SGC-7901 cells and enriched GCSCs. (*p < 0.05, **p < 0.01, ***p < 0.001).

The growth morphology of normal cultured and serum-free cultured GCSCs was assessed and the latter aggregated into clusters (**Figure 1A**). RT-qPCR results showed significantly higher stemness of CD44 and OCT4 in serum-free cultured GCSCs than in normal cultured SGC-7901 (**Figure 1B**). Western blot analysis also indicated consistent results (**Figure 1C**). This experiment revealed a successful culture of GCSCs (3) for subsequent experiments.

### 3.2 PGD2/PTGDR2 signaling inhibits self-renewal capacity of GCSCs

**Figure 2:**
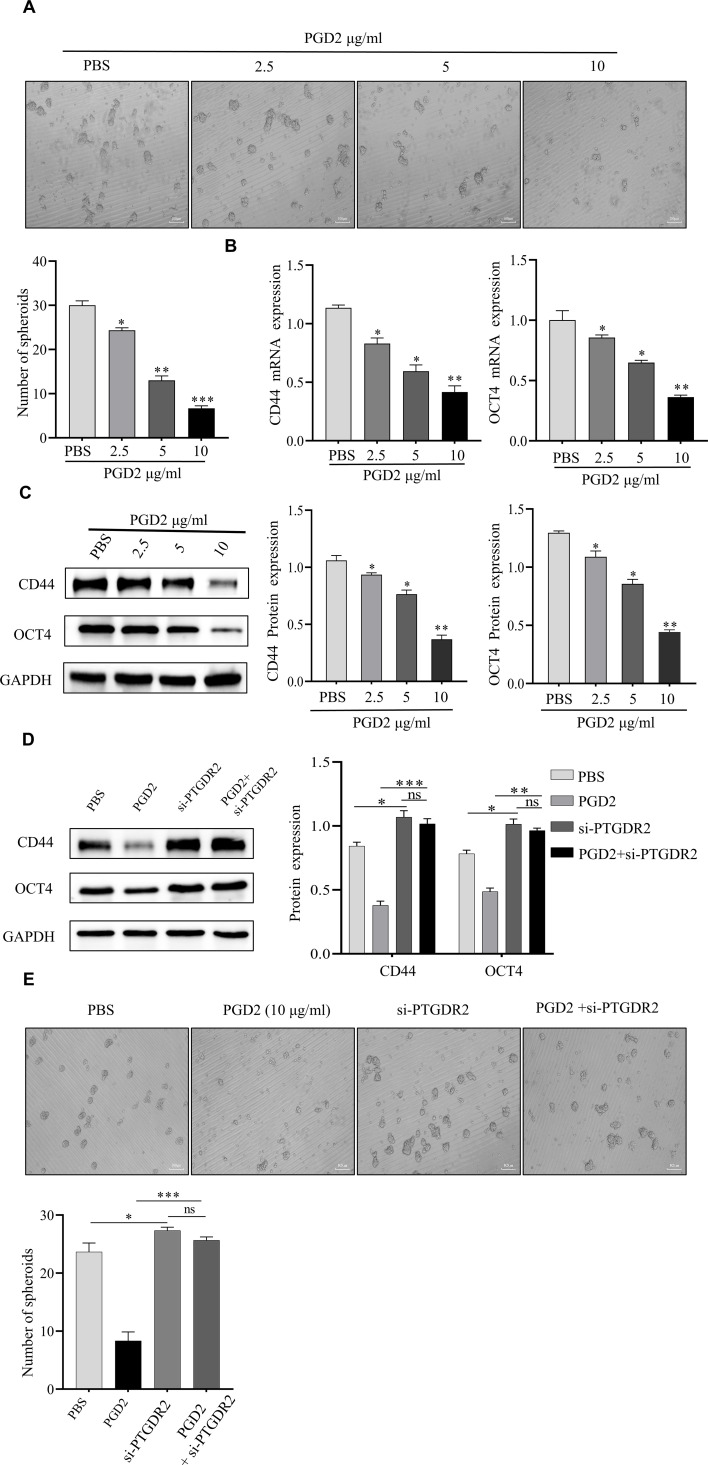
PGD2 depends on PTGDR2 expression and inhibits the self-renewal capacity of GCSCs in a concentration-dependent manner. **(A)** Sphere formation assay analyzed the sphere formation ability of GCSCs (SGC-7901) cells treated with different concentrations of PGD2. **(B, C)** Western blot and RT-qPCR analyses were performed to detect the expression of stemness-associated proteins in GCSCs treated with different concentrations of PGD2. **(D)** Western blot was performed to detect the effect of stemness in PTGDR2 knockdown GCSCs treated with PGD2 (10 μg/mL). **(E)** Sphere-forming assay to analyze the number and size of spheroplasts in PTGDR2 knockdown GCSCs treated with PGD2 (10 μg/mL). (*p < 0.05; **p < 0.01; ***p < 0.001; ns, not significant).

To investigate how PGD2/PTGDR2 signaling regulates the self-renewal capacity of GCSCs, GCSCs were treated with different concentrations of PGD2. Then, the effect of PGD2 on GCSCs was detected by sphere-forming assay, which showed that PGD2 inhibited GCSC’s sphere-forming ability in a concentration-dependent manner (**Figure 2A**). The expressions of CD44 and OCT4 stemness factors were assessed by Western blot and RT-qPCR, which revealed that PGD2 inhibited the protein and mRNA expression levels of CD44 and OCT4 in a concentration-dependent manner (**Figures 2B, C**). In addition, to elucidate the effect of PTGDR2 on the stemness of GCSCs, Western blot analysis was performed, which revealed that PTGDR2 knockout reduced tumor inhibition by PGD2 and increased GCSCs stemness (**Figure 2D**). Moreover, the PGD2-induced inhibition of the tumor’s sphere-forming ability was significantly attenuated (**Figure 2E**). Altogether, the above results indicated that the PGD2/PTGDR2 pathway could inhibit the self-renewal ability of GC and that the anti-tumor effect of PGD2 was dependent on the expression of PTGDR2.

### 3.3 PGD2 activates autophagy in GCSCs

**Figure 3:**
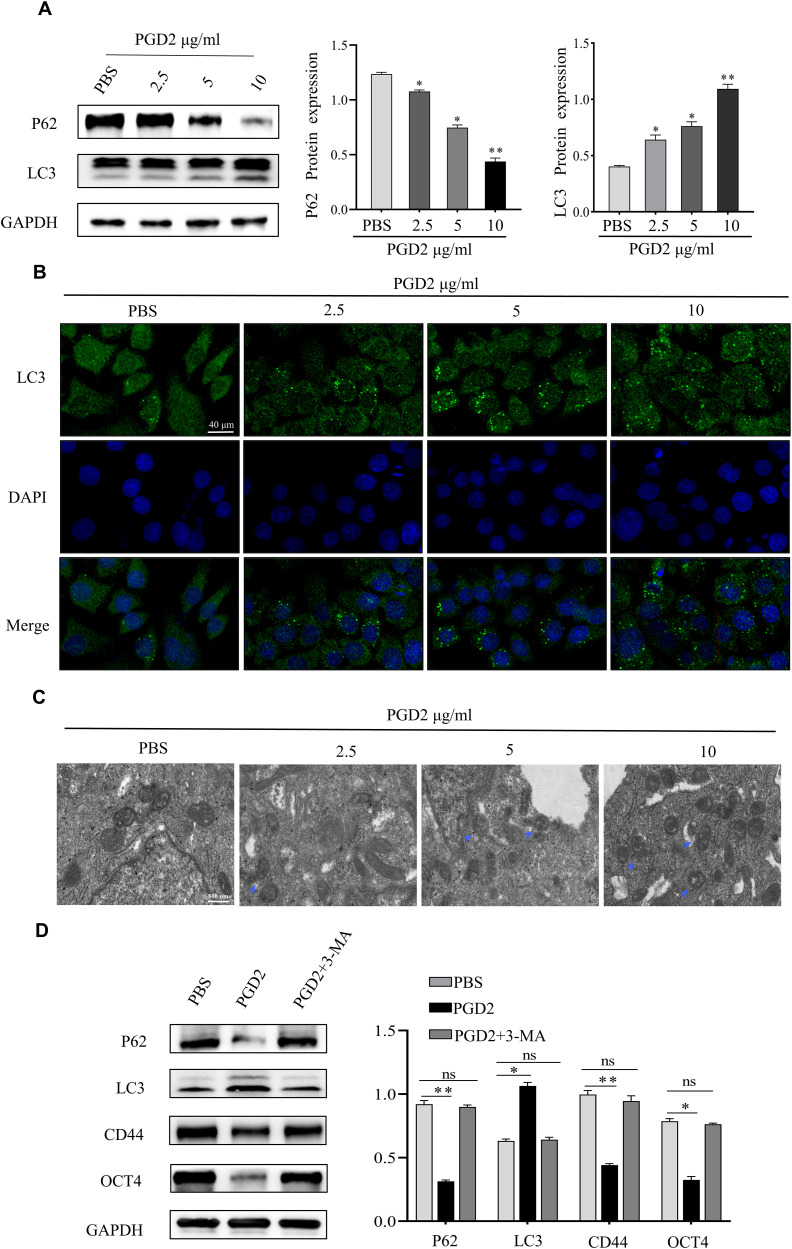
PGD2 stimulates the activation of autophagy in GCSCs. **(A)** Western blot detection of the expression of autophagy proteins in GCSCs after treatment with different concentrations of PGD2. **(B)** Confocal microscope observation of autophagic vesicle formation in GCSCs after treatment with PGD2. **(C)** Transmission electron microscope observation of autophagosome formation in GCSCs after treatment with PGD2. (blue arrows indicate the autophagy-lysosome structure); **(D)** Western blot detection of the expression of autophagy and stemness proteins after treatment with PGD2 (10 μg/mL) and 3-MA (10 μM). (*p < 0.05; **p < 0.01; ns, not significant).

In a previous study, Zhang et al. showed that STAT3 is an important regulator of CSCs, and PGD2 inhibited the expression of STAT3 (11). Furthermore, it has been indicated that STAT3 inhibition upregulates the expression of autophagy-related proteins, thereby activating autophagy (19). Since autophagy activation is critical for CSCs function, it was hypothesized that the PGD2/PTGDR2 signaling pathway may regulate GCSCs function by targeting autophagy activation. To indicate that PGD2 activates autophagy, GCSCs were treated with different concentrations of PGD2. The western blot analysis showed that PGD2 promoted LC3I to LC3II conversion and inhibited P62 protein expression in a concentration-dependent manner (**Figure 3A**). In addition, LC3 immunofluorescence revealed that PGD2 induced autophagic vesicle formation (**Figure 3B**). Consistently, transmission electron microscopy (TEM) indicated that PGD2 increases the number of autophagosomes in GCSCs (**Figure 3C**). Furthermore, to demonstrate the role of autophagy in GCSCs, whether PGD2-regulated autophagy affected the stemness of GC cells was determined. However, PGD2-induced autophagy was reversed and the stemness of GCSCs was inhibited after treatment with autophagy inhibitor 3-methyladenine (3-MA) (**Figure 3D**). Overall, PGD2 may regulate the malignant biological function of GCSCs by activating autophagy.

### 3.4 PGD2/PTGDR2 signaling cascade regulates ATG4B protein expression and stability

**Figure 4:**
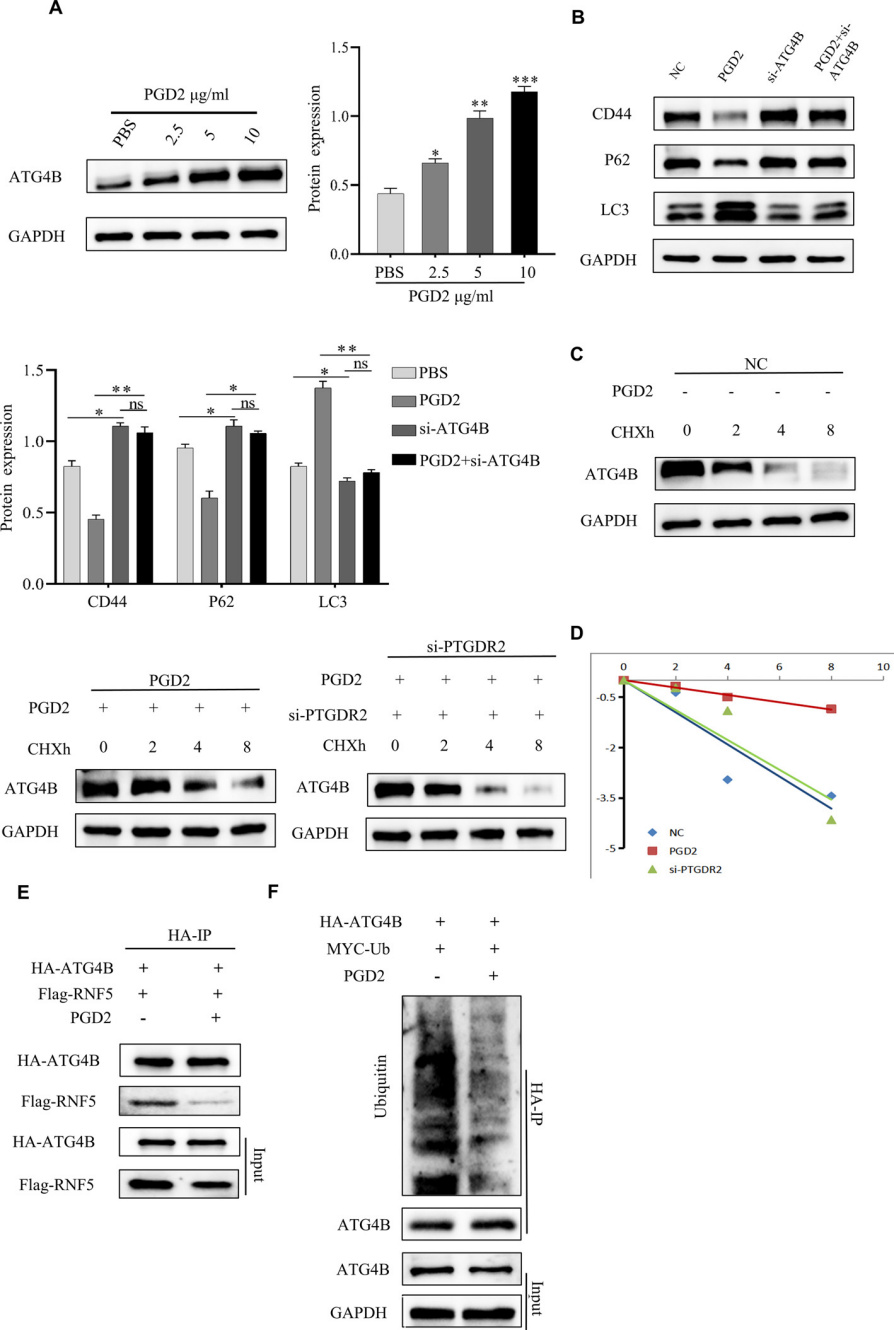
PGD2 promotes ATG4B protein expression and stability. **(A)** Western blot detection of ATG4B protein expression after treatment of SGC-7901 cells with different concentrations of PGD2. **(B)** Western blot detection of stemness and autophagy-related protein expression after ATG4B knockdown and PGD2 treatment in SGC-7901 cells. **(C)** After PGD2 (10 μg/mL) and cycloheximide(CHX) (100 μg/mL) addition, as well as PTGDR2 knockdown in SGC-7901 cells, respectively, the total proteins were extracted at different times, and the changes in ATG4B protein levels were subsequently detected by Western blot. **(D)** ATG4B protein abundance in **(C)** was quantified and plotted. **(E)** Immunoblot analysis of cells co-transfected with HA-ATG4B and Flag-RNF5 plasmid and treated with PGD2 (10 μg/mL), using indicated antibodies. **(F)** Immunoblot analysis of cells co-transfected with HA-ATG4B and MYC-Ub plasmid treated with PGD2 (10 μg/mL) and then MG-132 (10 μM) for 6 h, using indicated antibodies. ((*p < 0.05; **p < 0.01; ***p < 0.001; ns, not significant) cycloheximide(CHX) is an inhibitor of protein synthesis that blocks the translation process of proteins, MG-132 is a proteasome inhibitor that permeabilizes cells and selectively inhibits the proteasome.).

To further explore the molecular mechanism of PGD2 activation of autophagy, different concentrations of PGD2 were used to treat SGC-7901 cells. The results showed that the protein expression of ATG4B could be promoted under the stimulation of PGD2 (**Figure 4A**). Furthermore, the expression of relevant autophagy proteins after ATG4B knocking down was also assessed, which revealed that the expression of LC3 was down-regulated, while the expression of P62 was increased, suggesting that ATG4B plays an important role in PGD2-induced autophagy (**Figure 4B**). Moreover, ATG4B knockdown inhibited PGD2 suppression of stem cell stemness (**Figure 4B**). To stability test revealed that PGD2 treatment promoted the stability of ATG4B protein, which was significantly reduced after the knockdown of PTGDR2 (**Figures 4C, D**). In addition, the Co-IP experiment indicated that PGD2 treatment significantly reduced the binding of ATG4B to RNF5 (**Figure 4E**) as well as the levels of ATG4B ubiquitination (**Figure 4F**). The above results indicate that the expression and stability of ATG4B protein is regulated by PGD2/PTGDR2 signaling, suggesting that ATG4B plays an important role in PGD2 activation of autophagy.

### 3.5 PGD2 requires PTGDR2 for autophagy activation

**Figure 5:**
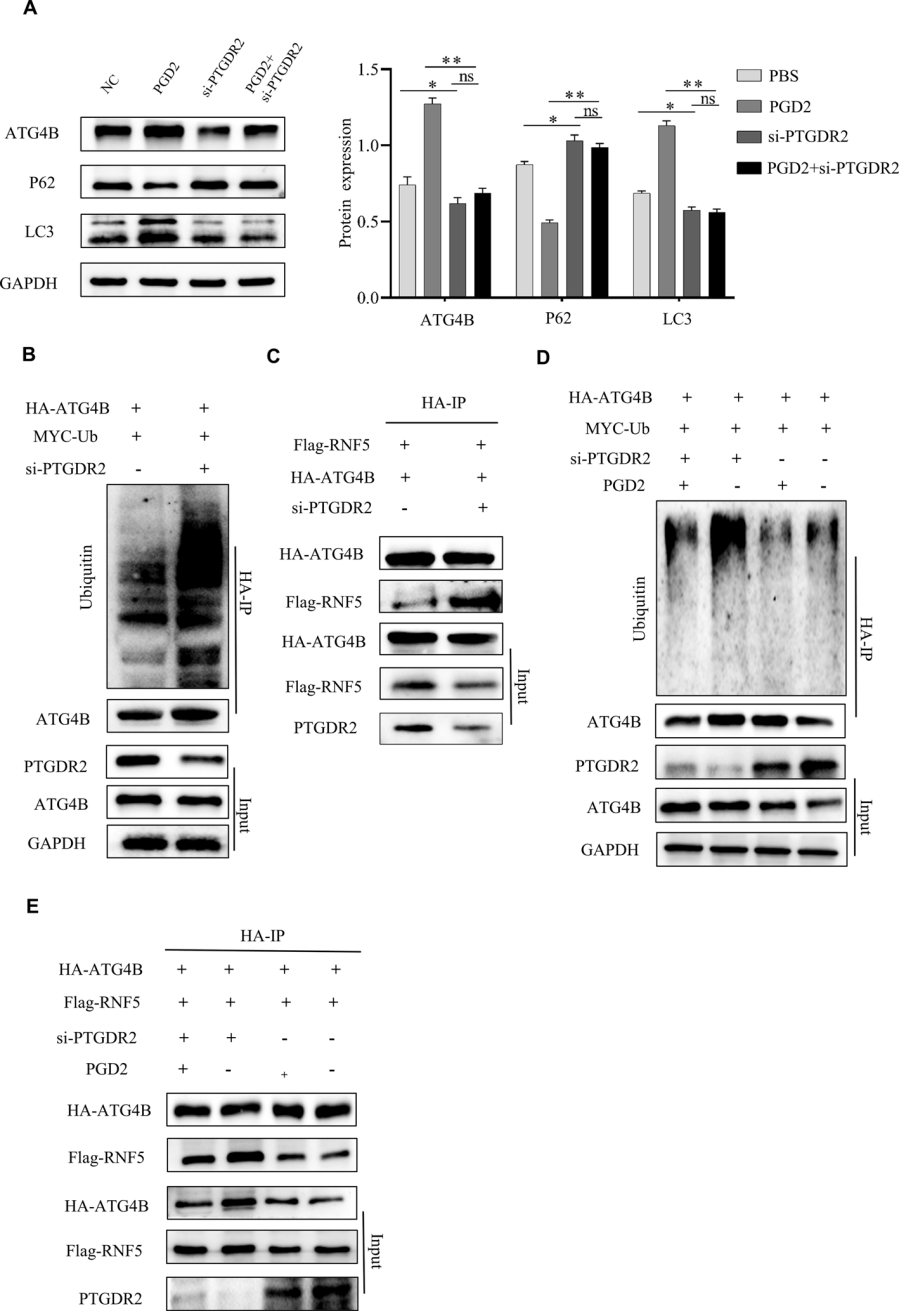
PGD2 requires PTGDR2 for autophagy activation. **(A)** Western blot analysis of autophagy-related protein expression in PTGDR2 knockdown and PGD2 treated SGC-7901 cells. **(B)** Immunoblot analysis of ATG4B and Ub expression in HA-ATG4B and MYC-Ub plasmids transfected PTGDR2 knockout cells. **(C)** Immunoblot analysis of HA-ATG4B and Flag-RNF5 transfected PTGDR2 knockout cells using indicated antibodies. **(D)** Immunoblot analysis of HA-ATG4B, MYC-Ub, and Flag-RNF5 plasmids transfected PTGDR2 knockout cells using indicated antibodies. **(E)** Immunoblot analysis of HA-ATG4B, Flag-RNF5 plasmids transfected, and PGD2 treated cells using indicated antibodies. (*p < 0.05; **p < 0.01; ns, not significant).

The Western blot experiments indicated that PTGDR2 knockdown blocked PGD2-induced autophagy, reduced LC3 expression, and reversed PGD2-reduced P62 expression (**Figure 5A**). whereas PTGDR2 knockdown increased the level of ATG4B ubiquitination and decreased ATG4B protein expression (**Figures 5A, B**). The results indicated that PTGDR2 is crucially involved in autophagy activation. Furthermore, the direct binding between RNF5 and ATG4B was also significantly enhanced after PTGDR2 knockdown (**Figure 5C**). Moreover, PTGDR2 knockdown increased the level of ATG4B ubiquitination and prevented PGD2-mediated reduction of ATG4B ubiquitination (**Figure 5D**). Thus, the interaction between RNF5 and ATG4B was regulated by PTGDR2 (**Figure 5E**). Overall, these data revealed that PTGDR2 is a key component of PGD2-induced autophagy activation.

### 3.6 PGD2 promotes PTGDR2 protein expression and enhances the competitive binding of PTGDR2 with RNF5, thereby affecting autophagy activation

**Figure 6:**
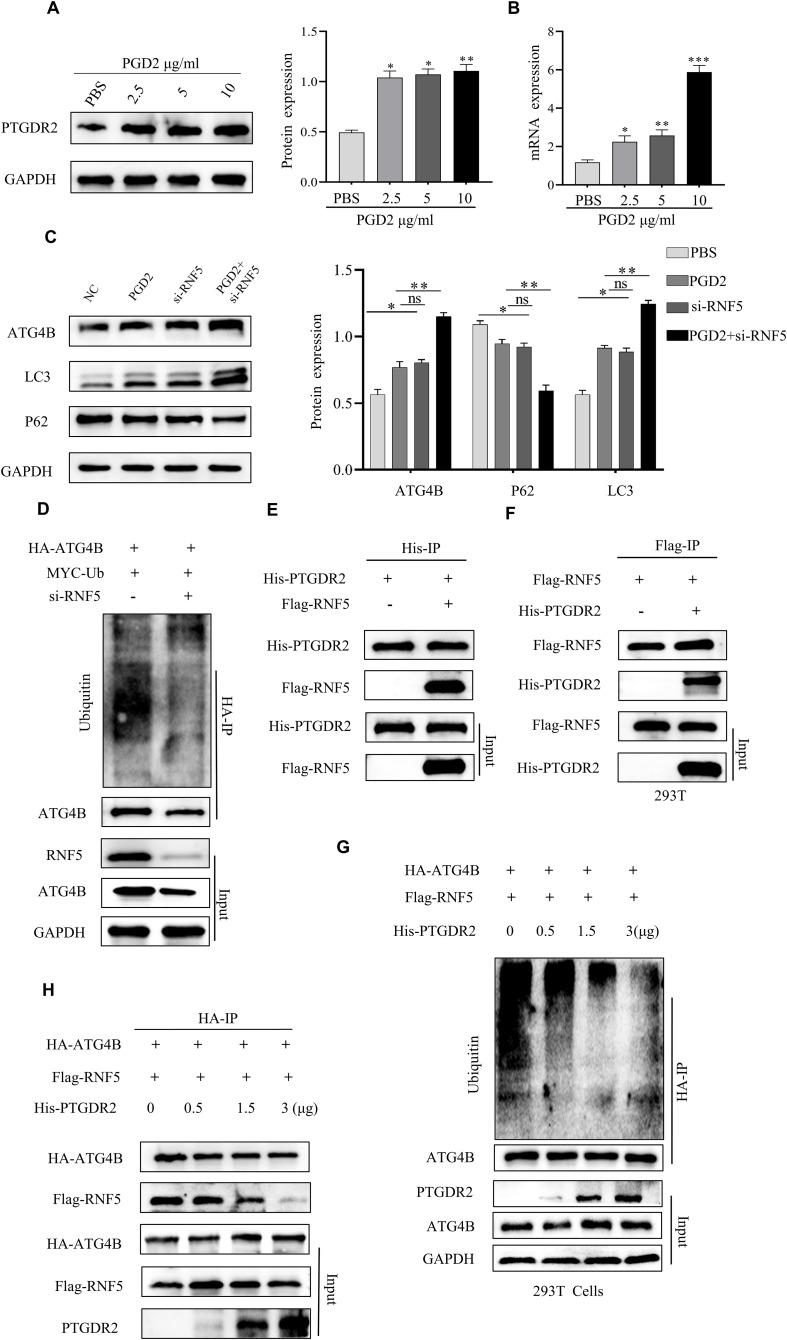
PGD2 induces PTGDR2 expression and promotes ATG4B ubiquitination. **(A, B)** Western blot and **(B)** RT-qPCR detection of PTGDR2 protein and mRNA expression, respectively, in SGC-7901 cells treated with different concentrations of PGD2. **(C)** Western blot detection of autophagy-related protein expression in RNF5 knockdown and PGD2 treated SGC-7901 cells. **(D)** Immunoblot analysis of cells transfected with HA-ATG4B, MYC-Ub, and knockdown RNF5 using indicated antibodies. **(E, F)** The expression of PTGDR2 and RNF5 in cell lysates, Flag-tagged RNF5 in protein complexes precipitated by the IP group, and His-tagged PTGDR2 were detected by reverse immunoprecipitation experiments using 293T cells. **(G)** Immunoblot analysis of 293T cells co-transfected with different concentrations of His-PTGDR2, HA-ATG4B (3 μg) plasmids, using indicated antibodies. **(H)** Immunoblot analysis of cells co-transfected with different concentrations of His-PTGDR2, HA-ATG4B, and Flag-RNF5 (3 μg) plasmids, using indicated antibodies. (*p < 0.05; **p < 0.01; ***p < 0.001; ns, not significant).

To further explore the molecular mechanism of autophagy, the western blot analysis was performed, which revealed that exogenous PGD2 promotes PTGDR2 expression (**Figure 6A**). Consistently, RT-qPCR assay revealed that different concentrations of PGD2 promote PTGDR2 mRNA expression after stimulation (**Figure 6B**). Kuang et al. showed that the E3 ubiquitin ligase RNF5 restricts the basal level of autophagy by regulating the stability of ATG4B and degrades it *via* the proteasomal pathway, thereby decreasing ATG4B expression and inhibiting autophagy activation (16). In addition, we found an increased expression of ATG4B protein abundance after stimulation of SGC-7901 and 293T cells by addition of PGD2, respectively, which was even more pronounced after addition of the MG132 inhibitor, suggesting that ATG4B may be degraded via the proteasomal pathway, which is in line with the studies of Kuang et al. (S a-d). Since ATG4B cleaves LC3 protein to LC3I, it plays an important role in autophagy. In addition, this study detected an increase in autophagy protein expression by western blot after RNF5 knockdown, which is consistent with the findings of Kuang et al. Here, it was observed that PGD2 stimulation promoted autophagy protein expression, which was increased after RNF5 knockdown followed by PGD2 stimulation (**Figure 6C**). Simultaneous knockdown of RNF5 inhibited ATG4B ubiquitination levels and increased protein expression thereby activating autophagy levels (**Figures 6C, D**). The above data suggests that RNF5 inhibits the expression of ATG4B protein and thus hinders the activation of autophagy. Other studies have shown an interaction between PTGDR2 and RNF5 (17). Here, the Co-IP experiments showed that Flag-tagged RNF5 was detected in the IP group. In addition, reverse immunoprecipitation experiments were also performed in the IP group with 293T cells, and His-tagged PTGDR2 (**Figures 6E, F**), which further demonstrated the interaction between PTGDR2 and RNF5. Moreover, because PTGDR2 and RNF5 can bind to each other, increased PTGDR2 expression can reduce the binding of RNF5 to ATG4B and inhibit the degradation of ATG4B through the proteasome pathway. Therefore, PTGDR2 overexpression was induced to different levels in 293T cells, which revealed that increased PTGDR2 expression gradually reduces ATG4B ubiquitination level (**Figure 6G**). In addition, with increasing PTGDR2 expression, the direct binding between RNF5 and ATG4B decreases (**Figure 6H**). Taken together, PGD2 activates the PTGDR2 downstream signaling cascade by inducing the expression of its receptor PTGDR2 and competes with ATG4B for binding RNF5 *via* PTGDR2, which reduces the binding of RNF5 to ATG4B, promotes ATG4B protein expression, and further activates autophagy to promote autophagic apoptosis in tumor cells.

## 4 Discussion

The CSCs are known as tumor-initiating cells, and their very small population can promote tumorigenesis, which causes tumor angiogenesis, invasiveness, drug resistance, and recurrence after treatment (20, 21). Many studies have demonstrated the presence of CSCs in various tumors and their association with the entire tumor developmental process, therefore, targeting CSCs may be the key to inhibiting tumor development (6). Vafaeinik et al. found that PGD2 inhibited epithelial-to-mesenchymal transition (EMT) in lung cancer A549 cell lines (22). The EMT has been indicated to be associated with the maintenance of stemness in CSCs (20). Moreover, the PGD2/PTGDR2 signaling pathway can inhibit the *in vitro* and *in vivo* invasive growth of GC cells; however, the specific molecular mechanisms regulating CSCs remain unknown. Therefore, this study elucidated how the PGD2/PTGDR2 signaling pathway activates autophagy *via* ATG4B ubiquitination to further regulate the biological functions of CSCs.

However, current research on PGD2 is mainly focused on its regulatory role in immune system diseases, such as inflammation and asthma (3, 21). PGD2 has anti-inflammatory properties and it inhibits vascular permeability in acute lung inflammation and a mouse tumor model (23, 24). Recent literature has indicated that PGD2 binding with its receptor can promote apoptosis as well as prevent GC cell proliferation and migration (3, 11). Furthermore, Pan et al. demonstrated that the PGD2/PTGDS signaling pathway inhibited MCF-7 cell proliferation and migration by decreasing TWIST 2 levels, and also suppressed the expression of ALDH1A1, a breast cancer stem cell marker, which repressed angiogenesis and self-renewal ability of breast cancer cells (25). Moreover, Qian et al. indicated that in acute myeloid leukemia, PTGDR2 inhibited the KRAS-mediated MAPK and PI 3 K/AKT/mTOR signaling pathways, thereby promoting apoptosis of LICs (26). Altogether, these studies indicated that the PGD2/PTGDR2 signaling pathway is significantly related to stem cell differentiation and self-renewal.

This study highlighted a novel mechanism of how the PGD2/PTGDR2 signaling cascade regulates autophagy and stemness in GCSCs. Although autophagy has been extensively studied in tumors, Autophagy is a double-edged sword in tumors, and it is generally believed that autophagy promotes tumor cell growth and maintains CSCs stemness (27, 28). Previous studies have shown that PGD2 inhibits STAT3 activation, and STAT3 inhibition promotes autophagy activation (19). Therefore, it was proposed that PGD2/PTGDR2 signaling could inhibit the stemness of GCSCs by activating autophagy. It was revealed that PGD2 induces the formation of autophagosomes in GCs, which activates autophagy and inhibits the stemness of their GCSCs. Furthermore, the inhibitory effect of PGD2 on stemness markers in GCSCs was reversed by treatment with the autophagy inhibitor 3-MA. Moreover, the autophagy protein ATG4B knockdown inhibited PGD2-mediated activation of autophagy and inhibition of GCSCs stemness, indicating that ATG4B plays an important role in activating autophagy. Therefore, it was suggested that the PGD2/PTGDR2 signaling cascade may influence the malignant biological function of GCSCs through autophagy.

To further explore the molecular mechanism of activated autophagy, western blot analysis was performed, which revealed that PGD2 could promote the expression and stability of ATG4B protein, and reduce the ubiquitination level of ATG4B. ATG4B is an autophagy protein that cleaves LC3 proteins to generate active LC3I. Sun recently showed that the E3 ubiquitin ligase UBE3C can regulate autophagy levels through K-33-mediated ATG4B ubiquitination without causing protein degradation (29). Furthermore, the E3 ubiquitin ligase RNF5 promotes ATG4B ubiquitination and degradation *via* the proteasomal pathway, thereby inhibiting the activation of autophagy (16), suggesting that the presence of RNF5 inhibits the level of autophagy. Both the above studies indicated that the level of autophagy can be affected by regulating the expression of the ATG4B protein, which further suggests the key role of ATG4B in autophagy activation. Roy et al. showed that PTGDR2, the ligand for PGD2, interacts with RNF5 (17). Here, it was indicated by a western blot that PGD2 promotes the protein expression of PTGDR2, whereas PTGDR2 knockdown inhibits PGD2-mediated activation of autophagy and suppression of stemness. In addition, Co-IP experiments demonstrated that PGD2 reduced the binding of RNF5 to ATG4B and decreased the level of ubiquitination of ATG4B, thereby promoting ATG4B protein expression. Moreover, ATG4B degradation was inhibited after the PTGDR2 overexpression plasmid at different concentrations was employed, which also indicated that ATG4B binds RNF5 and the level of ATG4B ubiquitination gradually decreases. Overall, these data suggest that PTGDR2 may promote ATG4B protein expression by competing with ATG4B for binding to RNF5 and reducing RNF5 degradation of ATG4B protein *via* the proteasome pathway. Altogether, the higher the PGD2 and PTGDR2 expression, the higher the ATG4B protein expression the and lower the RNF5 expression, which activates autophagy and causes autophagic apoptosis in GCSCs. The conclusion of this research is supported by three experimental results: (I) PGD2 reduces RNF5 and ATG4B interactions, and PTGDR2 knockdown enhances RNF5-ATG4B interactions and reverses the effects of PGD2 on autophagy and self-renewal capacity, (II) RNF5 knockdown reduces ATG4B ubiquitination and promotes autophagy, and (III) PGD2 promotes the expression of PTGDR2 and increased PTGDR2 expression reduces the binding between RNF5 and ATG4B, decreases ATG4B ubiquitination, and increases autophagy. Therefore, it was inferred that the PGD2/PTGDR2 signaling pathway may promote autophagic apoptosis in GCSCs by regulating the ubiquitination of ATG4B, which in turn activates autophagy. The mechanism involves PTGDR2 competing with ATG4B for binding to RNF5 and attenuating the direct binding of RNF5 to ATG4B to reduce the level of ATG4B ubiquitination, thereby promoting ATG4B protein expression as well as autophagy levels.

This study demonstrated a novel mechanism of PGD2 in inhibiting the stemness of GCSCs, explored how PGD2 inhibits the self-renewal of GCSCs by activating autophagy, and preliminary assessed the molecular mechanism of autophagy activation. The acquired data indicated that PGD2 can promote PTGDR2 expression and inhibit the stemness of GCSCs, whereas PTGDR2 knockdown reduces the inhibitory effect of PGD2 on GCSCs, suggesting that PGD2 and its ligands form a positive-negative control loop in the regulation of CSCs. Altogether, it is tentatively suggested that the PGD2/PTGDR2 signaling pathway may influence gastric cancer development by regulating the ubiquitination of ATG4B.

## 5 Conclusions

In summary, this study identified that the PGD2/PTGDR2 signaling cascade inhibits gastric carcinogenesis and showed a mechanism by which this signaling cascade affects the self-renewal capacity of GCSCs by modulating autophagy. These findings highlight that the PGD2/PTGDR2 signaling cascade may be a novel target for cancer therapy.

## Data Availability

The original contributions presented in the study are included in the article/**Supplementary Material**. Further inquiries can be directed to the corresponding author.

## References

[1] Rao X Zhang C Luo H Zhang J Zhuang Z Liang Z. Targeting gastric cancer stem cells to enhance treatment response. Cells. (2022) 11:2828. doi: 10.3390/cells11182828 36139403 PMC9496718

[2] Nio K Yamashita T Kaneko S. The evolving concept of liver cancer stem cells. Mol Cancer. (2017) 16:4. doi: 10.1186/s12943-016-0572-9 28137313 PMC5282887

[3] Zhang Q Wang F Huang Y Gao P Wang N Tian H. PGD2/PTGDR2 signal affects the viability, invasion, apoptosis, and stemness of gastric cancer stem cells and prevents the progression of gastric cancer. Combinatorial Chem High Throughput Screening. (2024) 27:933–46. doi: 10.2174/1386207326666230731103112 37526190

[4] Najafi M Farhood B Mortezaee K. Cancer stem cells (CSCs) in cancer progression and therapy. J Cell Physiol. (2019) 234:8381–95. doi: 10.1002/jcp.v234.6 30417375

[5] Bessède E Staedel C Acuña Amador LA Nguyen PH Chambonnier L Hatakeyama M. Helicobacter pylori generates cells with cancer stem cell properties via epithelial-mesenchymal transition-like changes. Oncogene. (2014) 33:4123–31. doi: 10.1038/onc.2013.380 24096479

[6] Li K Dan Z Nie YQ. Gastric cancer stem cells in gastric carcinogenesis, progression, prevention and treatment. World J Gastroenterol. (2014) 20:5420–6. doi: 10.3748/wjg.v20.i18.5420 PMC4017057 24833872

[7] Ponta H Sherman L Herrlich PA. CD44: from adhesion molecules to signalling regulators. Nat Rev Mol Cell Biol. (2003) 4:33–45. doi: 10.1038/nrm1004 12511867

[8] Brungs D Aghmesheh M Vine KL Becker TM Carolan MG Ranson M. Gastric cancer stem cells: evidence, potential markers, and clinical implications. J Gastroenterol. (2016) 51:313–26. doi: 10.1007/s00535-015-1125-5 26428661

[9] Biddle A Mackenzie IC. Cancer stem cells and EMT in carcinoma. Cancer Metastasis Rev. (2012). doi: 10.1007/s10555-012-9345-0 22302111

[10] Murata T Aritake K Matsumoto S Kamauchi S Nakagawa T Hori M. Prostagladin D2 is a mast cell-derived antiangiogenic factor in lung carcinoma. Proc Natl Acad Sci United States America. (2011) 108:19802–7. doi: 10.1073/pnas.1110011108 PMC3241753 22106279

[11] Zhang B Bie Q Wu P Zhang J You B Shi H. PGD2/PTGDR2 signaling restricts the self-renewal and tumorigenesis of gastric cancer. Stem Cells (Dayton Ohio). (2018) 36:990–1003. doi: 10.1002/stem.2821 29604141

[12] Miller DR Thorburn A. Autophagy and organelle homeostasis in cancer. Dev Cell. (2021) 56:906–18. doi: 10.1016/j.devcel.2021.02.010 PMC8026727 33689692

[13] Ojha R Bhattacharyya S Singh SK. Autophagy in cancer stem cells: A potential link between chemoresistance, recurrence, and metastasis. Biores Open Access. (2015) 4:97–108. doi: 10.1089/biores.2014.0035 26309786 PMC4497670

[14] Che S Wu S Yu P. Lupeol induces autophagy and apoptosis with reduced cancer stem-like properties in retinoblastoma via phosphoinositide 3-kinase/protein kinase B/mammalian target of rapamycin inhibition. J Pharm Pharmacol. (2022) 74:208–15. doi: 10.1093/jpp/rgab060 33836050

[15] Kuang E Qi J Ronai Z. Emerging roles of E3 ubiquitin ligases in autophagy. Trends Biochem Sci. (2013) 38:453–60. doi: 10.1016/j.tibs.2013.06.008 PMC3771342 23870665

[16] Kuang E Okumura CYM Sheffy-Levin S Varsano T Shu VC Qi J. Regulation of ATG4B stability by RNF5 limits basal levels of autophagy and influences susceptibility to bacterial infection. PloS Genet. (2012) 8:e1003007. doi: 10.1371/journal.pgen.1003007 23093945 PMC3475677

[17] Roy SJ Glazkova I Fréchette L Iorio-Morin C Binda C Pétrin D. Novel, gel-free proteomics approach identifies RNF5 and JAMP as modulators of GPCR stability. Mol Endocrinol (Baltimore Md.). (2013) 27:1245–66. doi: 10.1210/me.2013-1091 PMC5427950 23798571

[18] Rothe K Lin H Lin KBL Leung A Wang HM Malekesmaeili M. The core autophagy protein ATG4B is a potential biomarker and therapeutic target in CML stem/progenitor cells. Blood. (2014) 123:3622–34. doi: 10.1182/blood-2013-07-516807 24755409

[19] Li H Chen L Li JJ Zhou Q Huang A Liu WW. miR-519a enhances chemosensitivity and promotes autophagy in glioblastoma by targeting STAT3/Bcl2 signaling pathway. J Hematol Oncol. (2018) 11:70. doi: 10.1186/s13045-018-0618-0 29843746 PMC5975545

[20] Shibue T Weinberg RA. EMT, CSCs, and drug resistance: the mechanistic link and clinical implications. Nat Rev Clin Oncol. (2017) 14:611–29. doi: 10.1038/nrclinonc.2017.44 PMC5720366 28397828

[21] Kupczyk M Kuna P. Targeting the PGD2/CRTH2/DP1 signaling pathway in asthma and allergic disease: current status and future perspectives. Drugs. (2017) 77:1281–94. doi: 10.1007/s40265-017-0777-2 PMC5529497 28612233

[22] Vafaeinik F Kum HJ Jin SY Min DS Song SH Ha HK. Regulation of epithelial-mesenchymal transition of A549 cells by prostaglandin D2. Cell Physiol Biochem: Int J Exp Cell Physiol Biochem Pharmacol. (2022) 56:89–104. doi: 10.33594/000000506 35333485

[23] Iwanaga K Nakamura T Maeda S Aritake K Hori M Urade Y. Mast cell-derived prostaglandin D2 inhibits colitis and colitis-associated colon cancer in mice. Cancer Res. (2014) 74:3011–9. doi: 10.1158/0008-5472.CAN-13-2792 24879565

[24] Oguma T Asano K Ishizaka A. Role of prostaglandin D(2) and its receptors in the pathophysiology of asthma. Allergol International: Off J Japanese Soc Allergol. (2008) 57:307–12. doi: 10.2332/allergolint.08-RAI-0033 18946232

[25] Pan J Zhang L Huang J. Prostaglandin D2 synthase/prostaglandin D2/TWIST2 signaling inhibits breast cancer proliferation. Anti-Cancer Drugs. (2021) 32:1029–37. doi: 10.1097/CAD.0000000000001111 34232948

[26] Qian F Nettleford SK Zhou J Arner BE Hall MA Sharma A. Activation of GPR44 decreases severity of myeloid leukemia via specific targeting of leukemia initiating stem cells. Cell Rep. (2023) 42:112794. doi: 10.1016/j.celrep.2023.112794 37459233 PMC10428076

[27] Zhao R He B Bie Q Cao J Lu H Zhang Z. AQP5 complements LGR5 to determine the fates of gastric cancer stem cells through regulating ULK1 ubiquitination. J Exp Clin Cancer Res: CR. (2022) 41:322. doi: 10.1186/s13046-022-02532-w 36372898 PMC9661769

[28] Cao Y Luo Y Zou J Ouyang J Cai Z Zeng X. Autophagy and its role in gastric cancer. Clin Chim Acta Int J Clin Chem. (2019) 489:10–20. doi: 10.1016/j.cca.2018.11.028 30472237

[29] Sun C Chen Y Gu Q Fu Y Wang Y Liu C. UBE3C tunes autophagy via ATG4B ubiquitination. Autophagy. (2023) 20:645–58. doi: 10.1080/15548627.2023.2299514 PMC10936621 38146933

